# EMPDTA: An End-to-End Multimodal Representation Learning Framework with Pocket Online Detection for Drug–Target Affinity Prediction

**DOI:** 10.3390/molecules29122912

**Published:** 2024-06-19

**Authors:** Dingkai Huang, Jiang Xie

**Affiliations:** School of Computer Engineering and Science, Shanghai University, Shanghai 200444, China; dingkaihuang@shu.edu.cn

**Keywords:** drug–target affinity, pocket detection, multimodal representation learning, fine-tune

## Abstract

Accurately predicting drug–target interactions is a critical yet challenging task in drug discovery. Traditionally, pocket detection and drug–target affinity prediction have been treated as separate aspects of drug–target interaction, with few methods combining these tasks within a unified deep learning system to accelerate drug development. In this study, we propose EMPDTA, an end-to-end framework that integrates protein pocket prediction and drug–target affinity prediction to provide a comprehensive understanding of drug–target interactions. The EMPDTA framework consists of three main modules: pocket online detection, multimodal representation learning for affinity prediction, and multi-task joint training. The performance and potential of the proposed framework have been validated across diverse benchmark datasets, achieving robust results in both tasks. Furthermore, the visualization results of the predicted pockets demonstrate accurate pocket detection, confirming the effectiveness of our framework.

## 1. Introduction

Proteins are the workhorses in biological organisms, orchestrating virtually all biological processes. They often necessitate interactions with other molecules, termed ligands, to fulfill their specialized functions. Drug-like small molecules binding to proteins are important and widely studied, as they can facilitate drug discovery [1]. In most drug design projects, the initial goal is to find ligands that bind to a specific protein target with high affinity and specificity [2]. However, the costs associated with failed trials are substantial, with the median capitalized research and development investment to bring a new drug to market estimated at $985.3 million between 2009 and 2018 by the US Food and Drug Administration (FDA) [3]. Given these challenges, there is an urgent need for rapid screening of promising candidate drugs using computational methods. As such, binding pocket detection and drug–target affinity (DTA) prediction emerge as pivotal downstream tasks offering valuable insights for drug discovery.

Binding pocket detection plays a critical role in the initial stages of drug discovery. Traditional template-based and energy-based methods rely on high-quality templates or complex energy simulations, often encountering limitations with data and trade-offs in efficiency. Recently, machine learning methods leveraging protein geometric features have demonstrated remarkable performance and generalization capabilities [4]. In early geometric-based methods, Fpocket [5] treats atoms in proteins as spheres, calculates the alpha spheres of each atom, and derives the overall surface shape of protein molecules by merging and trimming adjacent alpha spheres. In contrast, P2Rank [6], another widely used tool, employs Connolly points [7] to represent solvent-reachable surfaces and model protein surfaces. After clustering the features, a random forest classifier distinguishes each Connolly point into two ligandable and unligandable categories. These detection methods are frequently utilized as standalone procedures in the drug development process [8].

After identifying binding pockets, assessing the drug–target affinity becomes pivotal in determining the strength of binding interactions. Methods for predicting drug–target affinity can be broadly categorized into similarity-based, sequence-based, and structure-based approaches. Similarity-based methods like KronRLS [9] and SimBoost [10] operate on the principle of guilt-by-association, assuming that similar drugs interact with similar targets and vice versa. However, these methods are often limited by knowledge constraints and struggle to generalize to novel scenarios. Sequence-based methods such as DeepDTA [11] and GraphDTA [12] follow the conventional approach of processing drug and target inputs separately. The primary distinction between DeepDTA and GraphDTA lies in whether they convert molecular compounds into graph structures for representation, as graphs offer a more suitable format for molecules. With the growing accumulation of protein structures and the advent of protein structure prediction models like AlphaFold2 [13], numerous structure-based methods have emerged, overcoming the limitations of sequence modal information. Molecular docking, a traditional yet effective method, offers good interpretability but suffers from computational inefficiency due to the sampling–scoring paradigm [14,15,16,17]. As a result, E(3)-equivariant graph neural networks have emerged, capable of directly generating molecular conformations without the need for extensive sampling [18,19].

While binding pockets significantly influence affinity, few approaches effectively integrate pocket information into drug–target affinity (DTA) prediction. DeepPS, for instance, explicitly leverages functional motifs extracted from protein amino acid sequences [20]. Furthermore, TANKBind [8] utilizes P2Rank as a preprocessing step to refine the interaction scope. However, none of these methods achieve end-to-end affinity prediction with online binding pocket identification. The external modules for pocket detection lead to increased training and inference complexity.

To overcome these constraints, we introduce EMPDTA, an end-to-end multimodal representation learning framework with pocket online detection for drug–target affinity prediction. EMPDTA combines pocket detection and affinity prediction seamlessly through three main components: pocket online detection (POD) module, multimodal representation learning (MRL) module, and joint training (JT) module. The POD module employs residue-level point cloud sampling on the protein surface and fast quasi-geodesic convolution layers in feature extraction. Meanwhile, the MRL module incorporates multimodal features encoded from sequence, structure, and surface information of drug–target pairs. In the JT module, the insights gained from fine-tuning on the POD module enhance the performance of the affinity on small datasets. We also compare our method with the state-of-the-art (SOTA) methods on the benchmark datasets. Our framework demonstrates excellent predictive performance across both tasks, with the effectiveness further confirmed through the visualization of pocket predictions.

## 2. Results

### 2.1. Pocket Detection Performance

In our first experiment, we exclusively leverage different modalities of protein information on the POD module, setting aside the contributions of other modules in our framework. To specifically assess the effectiveness of our POD module, we evaluate its performance across three benchmark datasets with pocket labels (notably, the proteins in Filtered Davis are a subset of those in Davis). The results presented in Table 1 indicate that all online methods outperform the commonly utilized offline method (P2Rank), showing a significant lead.

On the smaller datasets (Davis and KIBA), the CNN model, leveraging sequence modal information, demonstrated commendable performance across both AUROC and AUPRC metrics, with a mere 0.4 M parameters. Interestingly, the GCN model, based on graphs, exhibited inferior performance compared to CNN, even with double the parameters. Particularly noteworthy was the significant decline in AUPRC, suggesting a notable decrease in GCN prediction accuracy. This disparity may stem from the conventional GCN architecture’s lack of adaptability to relational graph inputs. However, GearNet, specially designed for protein relational graphs, did not encounter this limitation. Nonetheless, the exceptional classification performance of GearNet comes at the expense of parameters, nearly tenfold that of CNN. Notably, our surface-based POD module boasts the lowest parameters, achieving performance close to GearNet in AUROC, albeit with slightly lower AUPRC.

On the larger dataset (PDBbind), the predictive abilities of different models can be evaluated in a more realistic context. Our POD module stands out as a leader across both metrics. With robust predictive performance observed across various datasets and minimal parameter requirements, the POD module underscores its potential as an online plug-in module in the drug-discovery pipeline.

### 2.2. Multimodal Models Achieve Better Performance Than Single Modal Models

Our experiments demonstrate that integrating multiple modalities with the DNN predictor enhances affinity prediction performance. To isolate the impact of the POD module, we bypass the pocket detection process and use the pocket labels as ground truth. We then compare the performance across different modalities on the filtered Davis dataset (Table 2).

The sequence modality achieves excellent performance as a single-modal model; leveraging features obtained through PLM models proves effective for affinity prediction tasks, closely approaching the performance of multimodal approaches. However, upon concatenating features from multiple modalities, the model’s performance remains consistently high. Notably, incorporating protein surface information leads to a slight improvement, underscoring the role of diverse modalities in enhancing affinity prediction tasks.

### 2.3. Joint Training with Fine-Tuning Demonstrates Superior Performance

Considering that our model primarily consists of POD modules and MRL modules, there are several training methods available for the JT module. Experiments in this section aim to address this issue, including whether to fine-tune the POD module and whether to integrate BCE loss for pocket prediction. The first consideration arises from the limited number of proteins in some datasets. Therefore, pretraining the POD module on the PDBbind dataset, which contains a substantial number of protein structures, enables the acquisition of more generalized knowledge from protein structures. Subsequently, fine-tuning the DNN predictor on smaller datasets reduces the required memory and accelerates the training process. For instance, on an RTX 4070Ti 12G graphic card, the batch size for fine-tuning mode is 64, and the training time is 105s (first line in Table 3), while the batch size is halved and training time doubles under the normal mode (second line in Table 3).

On the other side, comparing the single-task (affinity values) and multi-tasks (affinity values and pocket labels), the results presented in Table 3 suggest that augmenting the label information of pockets not only enhances affinity prediction performance but also yields high-quality pocket prediction outcomes. Compared to the single task on affinity prediction, our EMPDTA model with a fine-tuned approach led to improvements of 5.8% and 155.7% in AUROC and AUPRC (first and third line). The same trend is observed in the normal method, which clearly shows that with the pocket label, the POD module can learn more realistic information and provide a more accurate guide in affinity prediction. This experiment underscores that leveraging multiple labels and fine-tuning the POD module can yield the most optimal training outcomes. We use this training mode in our subsequent experiments and will no longer differentiate between them.

### 2.4. Comparison with State-of-the-Art Methods

Our proposed EMPDTA demonstrates leading performance on small datasets such as Filtered Davis. As shown in Table 4, our method achieves an RMSE of 0.663, significantly outperforming the state-of-the-art method MDeePred. Moreover, EMPDTA also excels in CI and Spearman’s rank correlation, with improvements of 1.6% and 5.9% over the second-best method, respectively.

EMPDTA also performs effectively on the Davis and KIBA benchmark datasets. Table 5 presents a comparison between EMPDTA and existing baseline models. Our model achieves a leading MSE of 0.218 on the Davis dataset, outperforming all baselines. However, EMPDTA attains a CI of 0.891 and an r_m_^2^ of 0.689, which are slightly lower than the best-performing models. Due to the significant decrease in protein quantity, our framework exhibits a decline in the KIBA dataset, as the designed POD and JT modules rely on patterns from protein pockets. Poor performance on these two datasets also indicates that enhancing predictive performance in affinity prediction with limited protein data remains a challenge in our framework. Future work will focus on improving the framework’s effectiveness in scenarios with insufficient protein quantity.

Our framework demonstrates leading performance on the PDBbind dataset, which includes a greater number of proteins. This enhanced performance is attributed to the richness of protein structures, enabling more effective pocket information extraction for predicting affinity values. Compared to the current state-of-the-art method, TANKbind, our model slightly leads off in RMSE and MAE and is almost identical in the other two metrics (Table 6).

### 2.5. Model Interpretability on Both Affinity and Pocket Prediction

A major feature of our proposed framework is its ability to output high-quality pocket prediction results while simultaneously predicting affinity, supported by the strong integration of POD, MRL, and JT modules. Table 7 lists two types of evaluation indicators for the four benchmark datasets. The results show that our framework achieves high performance in pocket metrics while maintaining affinity prediction performance on par with SOTA methods.

**Figure 1:**
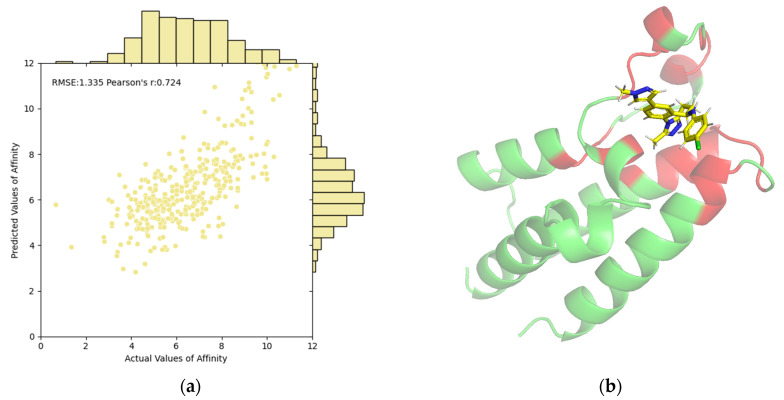
Visualization and interpretability of our EMPDTA on the PDBbind test set. (**a**) Correlations of predictive and actual values of affinity. (**b**) Predicted pocket (red) of complex 6K04 is close to the binding ligand (yellow) by PyMol. The non-pocket parts of the complex are in green.

The correlations between predictive values and ground truths on the PDBbind test set are illustrated in Figure 1a. The scatter points are predominantly clustered in a narrow, positively correlated region, further validating the predictive capability of our EMPDTA. Additionally, we have visualized the predicted pocket (red) and non-pocket (green) regions of the protein. For instance, Figure 1b displays the pocket detection results for the first complex in the test set (ID: 6K04). Our predicted pockets are spatially close to the binding ligand (name: CQF). By concentrating on both prediction tasks, the POD module accurately targets potential interaction areas. The multi-task prediction framework enhances the interpretability of results and streamlines the process by combining multiple predictions into a single, efficient step, thus reducing the need for traditional separate predictions.

## 3. Materials and Methods

### 3.1. Dataset Construction

Four benchmark datasets are compiled for model training and evaluation (Table 8). Given the integration of pocket detection and affinity prediction tasks in our framework, we opt to utilize these affinity benchmark datasets as the foundation, subsequently augmenting it by incorporating pocket labels for pocket detection.

- The Davis and Filtered Davis datasets. The Davis dataset comprises 30,056 drug–target pairs with affinity values ($pK_{d}$) among 72 drugs and 442 targets [24]. The Filtered Davis is derived from the Davis dataset, excluding pairs with no observed binding [21]. Consequently, the Filtered Davis dataset contains 72 drugs and 379 unique targets, forming 9125 interactions.
- KIBA dataset. KIBA incorporates a comprehensive combination of the inhibition constant ($K_{i}$), dissociation constant ($K_{d}$), and half-maximal inhibitory concentration (IC50) as affinity values [25]. It consists of 2111 drugs and 229 targets, forming 118,254 interactions.
- PDBbind dataset. PDBbind (v2020) comprises experimentally measured structures of 19,443 protein–ligand complexes with binding affinities [26].

As for pocket labels, the above sequence-based datasets (Davis, Filtered Davis, and KIBA) miss the information about the binding pockets. Therefore, we collect the pocket labels of the corresponding protein from the UniProt website (https://www.uniprot.org/, accessed on 1 May 2024). However, the structure-based PDBbind dataset conveniently offers structural files for protein pockets, facilitating their use as labels through straightforward index correspondence.

### 3.2. Problem Formulation

Given a drug–target pair with pocket labels and affinity values, our objective is twofold. Firstly, we aim to classify each residue of the protein as either “pocket” or “non-pocket,” treating this as a binary classification task [27]. Subsequently, utilizing the drug and online-extracted pocket features via multimodal encoders, the affinity value will be predicted by simply concatenating these features, framing it as a regression task [11].

### 3.3. Notation and Preprocessing

Consistent with our prior work, we represent the drug as an atom-level molecular graph $G_{m}=(V_{m},E_{m},R_{m})$, where edges signify chemical bonds. Utilizing the TorchDrug [28] implementation, we compute the drug node features, denoted by $V_{m}$. On the other hand, the protein is depicted as a residue-level graph $G_{p}=(V_{p},E_{p},R_{p})$, with each node possessing 3D coordinates $x\in R^{n\times 3}$ (the position of the alpha carbon of the residue). Subsequently, the edges $E_{p}$ are built based on seven types of $R_{p}$ representations including sequential, radius, and KNN edges following the GearNet [29]. The protein node features $V_{p}$ are computed using ESM-2b_650M to provide more biological knowledge [30].

### 3.4. Model Architecture of EMPDTA

**Figure 2:**
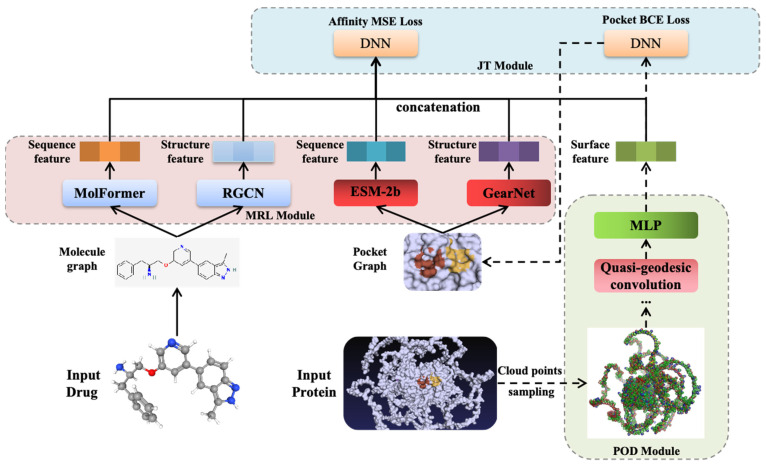
The proposed EMPDTA model with three modules. A. Pocket Online Detection Module. B. Multimodal Representation Learning Module. C. Joint Training Module.

The proposed EMPDTA model is illustrated in Figure 2. Our end-to-end model comprises three main modules conducted sequentially. Firstly, the pocket online detection module is employed for online sampling and detecting the binding pockets as the preprocessing stage (POD Module). Subsequently, multimodal features are extracted from drugs and pockets by multimodal encoders and fused through simple concatenation (MRL Module). Lastly, both pocket detection BCE loss (classification task) and affinity prediction MSE loss (regression task) are jointly considered during model training and testing (JT Module). Moreover, fine-tuning on POD module is proposed for accurate and quick training on datasets with small proteins. In the following sections, a brief description of each component will be provided.

### 3.5. Pocket Online Detection Module

Our pocket detection relies on the protein structures. In sequence-based datasets (Davis, Filtered Davis, and KIBA), all protein structures are obtained from the AlphaFold Protein Structure Database (https://alphafold.ebi.ac.uk/, accessed on 1 May 2024) using PDB ID mapping from the amino acid sequences. Moreover, the PDBbind dataset provides structurally resolved protein PDB files that can be directly utilized.

#### 3.5.1. Cloud Points Sampling

To enable online detection, we introduce a protein surface sampling method based on residue-level point clouds, eliminating the need for offline mesh generation. Inspired by dMaSIF [31], our sampling method extends the sampling objects from 6 types of atoms (C, H, O, N, S, Se) to 20 types of residues. Transitioning from atom-level to residue-level sampling can reduce the number of sampled points by nearly 20 times (each residue on average contains 20 atoms). Another distinction is the utilization of the Smooth Distance Function (*SDF*) in our protein surface definition (Formula (1)).

(1) $$SDF(x)=-\sigma (x)\cdot log\sum_{k=1}^{A}exp(-\|x-a_{k}\|/\sigma_{k})$$

The input is provided as a cloud of residues $\{a_{1},\ldots ,a_{A}\}\in R^{3}$, and $\left\|x-a_{k}\right\|$ represents the Euclidean distance between current point x and $a_{k}$. After a stable log-sum-exp reduction, a smoothing function σ(x) is used to form a reasonable protein surface. The radius of all residues can be seen in Table 9.

We sample the level set surface at radius *r* = 1.05 $\dot{A}$ by gradient descent via the loss function:

(2) $$E(x_{1},\ldots ,x_{N})=\frac{1}{2}\sum_{i=1}^{N}(SDF(x_{i})-r{)}^{2}$$

The sampled points ultimately fall on the surface of the protein after several iterations, and grid clustering is employed to ensure the density of the point cloud. Given the substantial number of distance calculation operations required for geodesic distance computation, PyKeOps is utilized to mitigate the issue of excessive graphics memory usage during distance matrix calculation [32].

#### 3.5.2. Quasi-Geodesic Convolution

Unlike convolution operations on images (CNN) and graphs (GCN), accurately defining convolution operations on curved surfaces remains challenging. Specifically, the Euclidean distance in three-dimensional space cannot accurately represent the surface distance between points. To maintain low computational costs, an approximate geodesic distance between two points $x_{i}$ and $x_{j}$ (with normal vector $n_{i},n_{j}$) on a surface is defined as:

(3) $$d_{ij}=\|x_{i}-x_{j}\|\cdot (2-\langle \hat{n_{i}},\hat{n_{j}}\rangle )$$

Therefore, the approximate geodesic distance $d_{ij}$ is not only determined by the Euclidean distance between two points but also by the local coordinate system determined by their respective normal vectors $p_{ij}$ [31]. Consequently, updated features that propagate along the surface can be obtained through the convolution operations using a local Gaussian window:

(4) $$f_{i}^{\prime }\leftarrow \sum_{j=1}^{N}Conv(x_{i},x_{j},f_{j})$$

**Figure 3:**
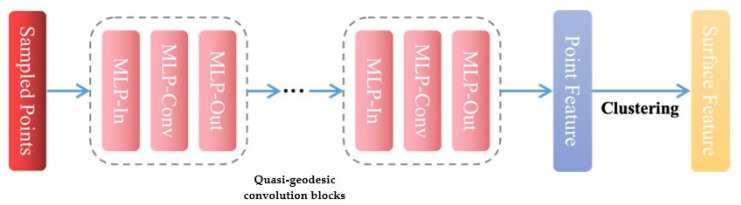
The structure of pocket online detection module.

Finally, a three-layer *MLP* with shared and learnable weights is employed as the quasi-geodesic convolution block to learn features in the local geodesic neighborhood of point *x_i_*. The convolution blocks update the point feature $f_{i}$ into $f_{i}^{\prime }$ as shown in Formula (5). The structure of the pocket detection module is illustrated in Figure 3.

(5) $$f_{i}^{\prime }\leftarrow \sum_{j=1}^{N}w(d_{ij})MLP(p_{ij})f_{j}$$

On one hand, surface features can be fed into a pocket classification DNN to predict the label of each residue (1 for pocket, 0 for non-pocket). On the other hand, these features can also be utilized for affinity prediction as a surface modality.

### 3.6. Multimodal Representation Learning Module

As validated in previous work, fused multimodal information helps to comprehensively understand drug–target interactions.

#### 3.6.1. Sequence Modality

The SMILES strings and amino acid sequences serve as primary representations, highlighting the fundamental components and functional modules of drugs and proteins, respectively. Notably, sequence information boasts vast quantities and convenient storage capabilities. Consequently, with the advancement of exceptional transformer-based pretrained language models (PLMs), numerous approaches aim to decipher the biological language. Through extensive pretraining on massive datasets, PLMs can extract a broad knowledge of functional regions within sequences. In our MRL module, two outstanding and prevalent PLMs, MolFormer [33] and ESM-2b [30], are chosen to extract features from the sequence modality. Although the benchmark dataset may not contain a large number of drugs and proteins, these features obtained through pretrained models can still provide rich information.

#### 3.6.2. Structure Modality

However, it is undeniable that the structural information of drugs and proteins offers a more micro perspective. The protein structure dictates its actual function and provides crucial geometric features such as folding states and binding sites, making it essential for understanding interactions and being widely employed in drug design. After preprocessing, relational graphs with multimodal information are constructed for both drugs and pockets. Graph neural network (GNN) models have proven effective in extracting topology representations of molecules [34,35]. A relational graph convolutional network [36] (RGCN) is further selected as the structure encoder for drugs to handle the four types of chemical bonds during message passing. Given the characteristics of pockets, GearNet [29] is chosen under the residue level of representation.

#### 3.6.3. Surface Modality

The surface features of proteins, serving as fingerprints of interactions, play a crucial role in understanding their dynamics. Therefore, we directly leverage the protein surface features extracted by our POD module rather than complex hand-crafted features. The module has also been proven effective in subsequent ablation experiments.

### 3.7. Joint Training Module

In our framework, we treat pocket prediction as a binary classification task for residues and affinity prediction as a regression task for drug–target pairs. Our JT module allows for a more cohesive integration between the predicted labels (pocket or non-pocket) and the pocket extraction. Specifically, we employ binary cross-entropy loss for the pocket detection classifier:

(6) $$L_{bce}=-\frac{1}{n_{p}}\sum_{j=1}^{n_{p}}\left[y_{j}log(p_{j})+(1-y_{j})log(1-p_{j})\right]$$

Regarding affinity, we employ the mean squared error (*MSE*) as the loss function, a common choice for regression tasks. Here, $P_{i}$ represents the prediction and $Y_{i}$ corresponds to the actual outputs, with n denoting the number of samples.

(7) $$MSE=\frac{1}{n}\sum_{i=1}^{n}{\left(P_{i}-Y_{i}\right)}^{2}$$

Therefore, the combined total loss consists of both the pocket classification loss and the affinity regression loss, with a weight factor β set to 0.5 in our experiments.

(8) $$L_{all}=L_{mse}+\beta L_{bce}$$

### 3.8. Model Training and Evaluation

EMPDTA is implemented using the PyTorch framework (https://pytorch.org/, accessed on 1 May 2024) and TorchDrug platform (https://torchdrug.ai/, accessed on 1 May 2024). AdamW [37] is utilized to update the model parameters. The hyperparameters for EMPDTA are determined through a grid search using weights and biases (https://wandb.ai/, accessed on 1 May 2024). Experiments are conducted using a workstation with two Intel Xeon Silver 4314 processors @ 2.40 GHz and dual NVIDIA RTX4090 GPU running on Linux.

We have listed eight state-of-the-art deep learning methods for DTA prediction on sequence-based benchmark datasets:

- KronRLS [9] utilizes two independent kernel functions to process molecular fingerprint similarities and the Smith–Waterman [38] score of targets.
- SimBoost [10] leverages features of drugs, targets, and drug–target pairs, using gradient-boosting regression trees as the prediction model.
- CGKronRLS [39,40] is a similarity-based method employing similarity matrices of drugs and targets with a kernel method for affinity prediction.
- DeepDTA [11] is an innovative method using two branches of CNN blocks to encode drug SMILES strings and protein sequences.
- MDeePred [21] feeds multi-channel protein features into a CNN and fingerprint-based molecule vectors into a fully connected neural network (FNN).
- GraphDTA [12] introduces molecular graphs into DTA prediction, marking a pioneering approach.
- DeepGLSTM [22] employs three blocks of graph convolutional networks (GCN) for drug molecules and bidirectional LSTM for protein sequences.
- MFR-DTA [23] proposes a novel architecture that includes BioMLP/CNN blocks, an Elem-feature fusion block, and a Mix–Decoder block to extract drug–target interaction (DTI) information and predict binding regions simultaneously.

The experimental results of the methods mentioned above are obtained from their respective papers. For the Davis and KIBA datasets, we use the split indexes provided in DeepDTA, allowing us to maintain the same train/validation/test sets. The split indexes for the Filtered Davis dataset are sourced from MDeePred. For the PDBbind dataset, the split is taken from TANKBind.

## 4. Conclusions

Understanding and identifying binding pockets and the affinity of drug–target interactions play a crucial role in drug development. Early virtual screening methods could quickly identify drugs with high-affinity values, but these values alone could not distinguish binding pockets due to the diversity of pockets. In this paper, a comprehensive prediction framework that incorporates pocket online detection, enabling the simultaneous prediction of drug–target affinity and binding pocket regions, is proposed.

Our model seamlessly integrates pocket online detection, multimodal representation learning, and joint training modules. The outstanding performance of our multitask and multimodal framework for two downstream tasks has been validated across benchmark datasets. Visualizing the binding pocket results revealed a high consistency between the predicted and actual binding pockets. This accurate prediction of affinity, coupled with the precise identification of binding pockets, offers a robust solution for future drug screening and opens new areas for exploration in drug development.

## Figures and Tables

**Table 1:** Predictive performance of different models on pocket prediction task.

Method	Davis		KIBA		PDBbind		Params
	AUROC↑	AUPRC↑	AUROC↑	AUPRC↑	AUROC↑	AUPRC↑	
P2Rank	0.8455	0.2055	0.8606	0.2290	0.6236	0.3749	offline
CNN	0.9344	0.7695	0.9320	0.6944	0.7996	0.5615	0.4 M
GCN	0.9148	0.5377	0.9144	0.5332	0.8065	0.5413	0.8 M
GearNet	**0.9448**	**0.7948**	**0.9513**	**0.8197**	0.8919	0.6838	4.6 M
POD	0.9427	0.6667	0.9351	0.6454	**0.9** **116**	**0.** **7085**	**0.03 M**

Note: The symbol “↑” means the bigger the better in this metric. The best result is in bold.

**Table 2:** Affinity performance of single-modal and multimodal models on Filtered Davis dataset.

Modal	Drug		Target			Affinity Metrics		
	Seq	Str	Seq	Str	Sur	RMSE↓	CI↑	Spearman↑
Single	√	—	√	—	—	0.469	0.746	0.524
	—	√	—	√	—	0.529	0.732	0.467
Multi	√	√	√	√	—	0.458	0.749	0.533
	√	√	√	√	√	**0.454**	**0.750**	**0.543**

Note: The symbol “↑” means the bigger the better in this metric, “↓” means the opposite. The best result is in bold.

**Table 3:** Training method comparison on Filtered Davis.

Training Method		Affinity Metrics			Pocket Metrics		Time↓ *
		RMSE↓	CI↑	Spearman↑	AUROC↑	AUPRC↑	
Single	Fine-tune	0.667 (0.010)	0.751 (0.005)	0.679 (0.011)	0.911 (0.004)	0.314 (0.011)	**105 s**
Single	Normal	0.675 (0.005)	0.746 (0.004)	0.667 (0.009)	0.519 (0.129)	0.039 (0.016)	236 s
Multi	Fine-tune	**0.6** **63** **(0.00** **6** **)**	**0.7** **52** **(0.00** **3** **)**	**0.6** **81** **(0.0** **08** **)**	0.964 (0.003)	**0.803** **(0.007)**	175 s
Multi	Normal	0.673 (0.007)	0.747 (0.002)	0.670 (0.004)	**0.971** **(0.007)**	0.763 (0.050)	224 s

Note: The symbol “↑” means the bigger the better in this metric, “↓” means the opposite. The best result is in bold. The standard deviations are given in parenthesis. * The time shows the average runtime per epoch on RTX4070Ti 12G.

**Table 4:** Performance comparison on Filtered Davis dataset.

Method	RMSE↓	CI↑	Spearman↑
CGKronRLS ^a^	0.769 (0.010)	0.740 (0.003)	0.643 (0.008)
MDeePred ^a^	0.742 (0.009)	0.733 (0.004)	0.618 (0.009)
DeepDTA ^a^	0.931 (0.015)	0.653 (0.005)	0.430 (0.013)
EMPDTA	**0.6** **63 (0.00** **6** **)**	**0.7** **52 (0.00** **3** **)**	**0.6** **81 (0.0** **08** **)**

Note: ^a^ These results are taken from MDeePred [21]. The symbol “↑” means the bigger the better in this metric, “↓” means the opposite. The best result is in bold. The standard deviations are given in parenthesis.

**Table 5:** Performance comparison on Davis and KIBA datasets.

Method	Davis			KIBA		
	MSE↓	CI↑	rm2↑	MSE↓	CI↑	rm2↑
KronRLS [9]	0.379	0.871	0.407	0.411	0.782	0.342
SimBoost [10]	0.282	0.872	0.644	0.222	0.836	0.629
DeepDTA [11]	0.261	0.878	0.630	0.194	0.863	0.673
GraphDTA [12]	0.229	0.893	—	0.147	0.882	—
DeepGLSTM [22]	0.232	0.895	0.680	**0.133**	0.897	**0.792**
MFR-DTA [23]	0.221	**0.905**	**0.705**	0.136	**0.898**	0.789
EMPDTA	**0.218**	0.891	0.689	0.142	0.878	0.763

Note: The symbol “↑” means the bigger the better in this metric, “↓” means the opposite. The best result is in bold. The standard deviations are given in parenthesis if available. — These results are not reported from original studies.

**Table 6:** A comparison of different methods on PDBbind v2020.

Methods	RMSE↓	Pearson↑	Spearman↑	MAE↓
TransCPI ^b^	1.741	0.576	0.540	1.404
MONN ^b^	1.438	0.624	0.589	1.143
PIGNet ^b^	2.640	0.511	0.489	2.110
IGN ^b^	1.433	0.698	0.641	1.169
HOLOPROT ^b^	1.546	0.602	0.571	1.208
STAMPDPI ^b^	1.658	0.545	0.411	1.325
TANKBind ^b^	1.346	**0.726**	**0.703**	1.070
EMPDTA	**1.335**	0.724	0.698	**1.069**

Note: ^b^ These results are taken from TANKBind [8]. The symbol “↑” means the bigger the better in this metric, “↓” means the opposite. The best result is in bold.

**Table 7:** Our EMPDTA performance on benchmark datasets.

Dataset	Affinity Metrics			Pocket Metrics	
	RMSE↓	CI↑	Spearman↑	AUROC↑	AUPRC↑
Filtered Davis	0.663	0.752	0.681	0.964	0.803
Davis	0.468	0.891	0.701	0.987	0.876
KIBA	0.385	0.871	0.865	0.964	0.686
PDBbind	1.335	0.755	0.698	0.874	0.581

Note: The symbol “↑” means the bigger the better in this metric, “↓” means the opposite.

**Table 8:** Summary of the benchmark datasets.

Datasets	Affinity-Related			Pocket-Related	
	Drugs	Targets	Interactions	Pocket Ration	Average residues
Davis	68	442	30,056	1.36%	790
Filtered Davis	68	379	9125	1.36%	790
KIBA	2111	229	118,254	1.79%	729
PDBbind *	15,689	12,828	19,350	9.51%	692

Note *: Due to preprocessing reasons, 19,350 out of 19,443 in PDBbind are gathered.

**Table 9:** Radius statistics of 20 different protein residues.

name	GLY	ALA	THR	SER	CYS	ASN	ASP	PRO	GLN	VAL
Radius	1.46	1.54	1.61	1.64	1.80	1.83	1.88	1.88	1.96	1.99
name	ILE	LEU	HIS	MET	GLU	LYS	ARG	PHE	TYR	TRP
Radius	1.99	2.02	2.02	2.05	2.07	2.17	2.17	2.18	2.19	2.38

## Data Availability

The source data and code repository can be accessed at https://github.com/BioCenter-SHU/EMPDTA, accessed on 1 May 2024.

## References

[1] Pei Q., Gao K., Wu L., Zhu J., Xia Y., Xie S., Qin T., He K., Liu T.-Y., Yan R. (2023). FABind: Fast and Accurate Protein-Ligand Binding. Adv. Neural Inf. Process. Syst..

[2] Dhakal A., McKay C., Tanner J.J., Cheng J. (2022). Artificial Intelligence in the Prediction of Protein–Ligand Interactions: Recent Advances and Future Directions. Brief. Bioinform..

[3] Wouters O.J., McKee M., Luyten J. (2020). Estimated Research and Development Investment Needed to Bring a New Medicine to Market, 2009–2018. JAMA.

[4] Stank A., Kokh D.B., Fuller J.C., Wade R.C. (2016). Protein Binding Pocket Dynamics. Acc. Chem. Res..

[5] Le Guilloux V., Schmidtke P., Tuffery P. (2009). Fpocket: An Open Source Platform for Ligand Pocket Detection. BMC Bioinform..

[6] Krivák R., Hoksza D. (2018). P2Rank: Machine Learning Based Tool for Rapid and Accurate Prediction of Ligand Binding Sites from Protein Structure. J. Cheminform..

[7] Huang B., Schroeder M. (2006). LIGSITEcsc: Predicting Ligand Binding Sites Using the Connolly Surface and Degree of Conservation. BMC Struct. Biol..

[8] Lu W., Wu Q., Zhang J., Rao J., Li C., Zheng S. (2022). TANKBind: Trigonometry-Aware Neural NetworKs for Drug-Protein Binding Structure Prediction. Adv. Neural Inf. Process. Syst..

[9] Pahikkala T., Airola A., Pietilä S., Shakyawar S., Szwajda A., Tang J., Aittokallio T. (2015). Toward More Realistic Drug-Target Interaction Predictions. Brief Bioinform..

[10] He T., Heidemeyer M., Ban F., Cherkasov A., Ester M. (2017). SimBoost: A Read-across Approach for Predicting Drug–Target Binding Affinities Using Gradient Boosting Machines. J. Cheminformatics.

[11] Öztürk H., Olmez E., Özgür A. (2018). DeepDTA: Deep Drug–Target Binding Affinity Prediction. Bioinformatics.

[12] Nguyen T., Le H., Quinn T.P., Nguyen T., Le T.D., Venkatesh S. (2021). GraphDTA: Predicting Drug–Target Binding Affinity with Graph Neural Networks. Bioinformatics.

[13] Jumper J., Evans R., Pritzel A., Green T., Figurnov M., Ronneberger O., Tunyasuvunakool K., Bates R., Žídek A., Potapenko A. (2021). Highly Accurate Protein Structure Prediction with AlphaFold. Nature.

[14] Trott O., Olson A.J. (2010). AutoDock Vina: Improving the Speed and Accuracy of Docking with a New Scoring Function, Efficient Optimization, and Multithreading. J. Comput. Chem..

[15] Ackloo S., Al-awar R., Amaro R.E., Arrowsmith C.H., Azevedo H., Batey R.A., Bengio Y., Betz U.A.K., Bologa C.G., Chodera J.D. (2022). CACHE (Critical Assessment of Computational Hit-Finding Experiments): A Public–Private Partnership Benchmarking Initiative to Enable the Development of Computational Methods for Hit-Finding. Nat. Rev. Chem..

[16] Li X., Jacobson M.P., Friesner R.A. (2004). High-Resolution Prediction of Protein Helix Positions and Orientations. Proteins: Struct. Funct. Bioinform..

[17] Gentile F., Agrawal V., Hsing M., Ton A.-T., Ban F., Norinder U., Gleave M.E., Cherkasov A. (2020). Deep Docking: A Deep Learning Platform for Augmentation of Structure Based Drug Discovery. ACS Cent. Sci..

[18] Batzner S., Musaelian A., Sun L., Geiger M., Mailoa J.P., Kornbluth M., Molinari N., Smidt T.E., Kozinsky B. (2022). E(3)-Equivariant Graph Neural Networks for Data-Efficient and Accurate Interatomic Potentials. Nat. Commun..

[19] Roche R., Moussad B., Shuvo M.H., Bhattacharya D. (2023). E(3) Equivariant Graph Neural Networks for Robust and Accurate Protein-Protein Interaction Site Prediction. PLoS Comput. Biol..

[20] D’Souza S., Prema K.V., Balaji S., Shah R. (2023). Deep Learning-Based Modeling of Drug–Target Interaction Prediction Incorporating Binding Site Information of Proteins. Interdiscip. Sci. Comput. Life Sci..

[21] Rifaioglu A.S., Cetin Atalay R., Cansen Kahraman D., Doğan T., Martin M., Atalay V. (2021). MDeePred: Novel Multi-Channel Protein Featurization for Deep Learning-Based Binding Affinity Prediction in Drug Discovery. Bioinformatics.

[22] Mukherjee S., Ghosh M., Basuchowdhuri P. (2022). DeepGLSTM: Deep Graph Convolutional Network and LSTM Based Approach for Predicting Drug-Target Binding Affinity. Proceedings of the 2022 SIAM International Conference on Data Mining (SDM) Proceedings.

[23] Hua Y., Song X., Feng Z., Wu X. (2023). MFR-DTA: A Multi-Functional and Robust Model for Predicting Drug-Target Binding Affinity and Region. Bioinformatics.

[24] Davis M.I., Hunt J.P., Herrgård S., Ciceri P., Wodicka L., Pallares G., Hocker M., Treiber D.K., Zarrinkar P. (2011). Comprehensive Analysis of Kinase Inhibitor Selectivity. Nat. Biotechnol..

[25] Tang J., Szwajda A., Shakyawar S., Xu T., Hintsanen P., Wennerberg K., Aittokallio T. (2014). Making Sense of Large-Scale Kinase Inhibitor Bioactivity Data Sets: A Comparative and Integrative Analysis. J. Chem. Inf. Model..

[26] Liu Z., Su M., Han L., Liu J., Yang Q., Li Y., Wang R. (2017). Forging the Basis for Developing Protein-Ligand Interaction Scoring Functions. Acc. Chem. Res..

[27] Abdollahi N., Tonekaboni S., Huang J.J.C., Wang B., MacKinnon S. (2023). NodeCoder: A Graph-Based Machine Learning Platform to Predict Active Sites of Modeled Protein Structures. arXiv.

[28] Zhu Z., Shi C., Zhang Z., Liu S., Xu M., Yuan X., Zhang Y., Chen J., Cai H., Lu J. (2022). TorchDrug: A Powerful and Flexible Machine Learning Platform for Drug Discovery. arXiv.

[29] Zhang Z., Xu M., Jamasb A., Chenthamarakshan V., Lozano A., Das P., Tang J. (2022). Protein Representation Learning by Geometric Structure Pretraining. arXiv.

[30] Madani A., Krause B., Greene E.R., Subramanian S., Mohr B.P., Holton J.M., Olmos J.L., Xiong C., Sun Z.Z., Socher R. (2023). Large Language Models Generate Functional Protein Sequences across Diverse Families. Nat. Biotechnol..

[31] Sverrisson F., Feydy J., Correia B.E., Bronstein M.M. Fast End-to-End Learning on Protein Surfaces. Proceedings of the 2021 IEEE/CVF Conference on Computer Vision and Pattern Recognition (CVPR).

[32] Charlier B., Feydy J., Glaunès J., Collin F.-D., Durif G. (2020). Kernel Operations on the GPU, with Autodiff, without Memory Overflows. J. Mach. Learn. Res..

[33] Ross J., Belgodere B., Chenthamarakshan V., Padhi I., Mroueh Y., Das P. (2022). Large-Scale Chemical Language Representations Capture Molecular Structure and Properties. Nat. Mach. Intell..

[34] Klicpera J., Groß J., Günnemann S. (2020). Directional Message Passing for Molecular Graphs. arXiv.

[35] Li S., Zhou J., Xu T., Dou D., Xiong H. (2021). GeomGCL: Geometric Graph Contrastive Learning for Molecular Property Prediction. Proc. AAAI Conf. Artif. Intell..

[36] Schlichtkrull M., Kipf T., Bloem P., van den Berg R., Titov I., Welling M. (2018). Modeling Relational Data with Graph Convolutional Networks. Proceedings of The Semantic Web: 15th International Conference, ESWC 2018, Proceedings 15, Heraklion, Crete, Greece, 3–7 June 2018.

[37] Loshchilov I., Hutter F. Decoupled Weight Decay Regularization 2019. Proceedings of the 7th International Conference on Learning Representations.

[38] Smith T.F., Waterman M.S. (1981). Identification of Common Molecular Subsequences. J. Mol. Biol..

[39] Airola A., Pahikkala T. (2018). Fast Kronecker Product Kernel Methods via Generalized Vec Trick. IEEE Trans. Neural Netw. Learn. Syst..

[40] Cichońska A., Ravikumar B., Allaway R.J., Wan F., Park S., Isayev O., Li S., Mason M., Lamb A., Tanoli Z. (2021). Crowdsourced Mapping of Unexplored Target Space of Kinase Inhibitors. Nat. Commun..

